# Safety and effectiveness of the Canadian food ladders for children with IgE-mediated food allergies to cow’s milk and/or egg

**DOI:** 10.1186/s13223-023-00847-7

**Published:** 2023-11-06

**Authors:** Alanna Chomyn, Edmond S. Chan, Joanne Yeung, Scott Cameron, Gilbert T. Chua, Timothy K. Vander Leek, Brock A Williams, Lianne Soller, Elissa M. Abrams, Raymond Mak, Tiffany Wong

**Affiliations:** 1 Department of Pediatrics, Division of Clinical Immunology & Allergy, University of British Columbia, BC Children’s Hospital, Room 1C31B 4480 Oak Street, V5Z 4H4 Vancouver, BC Canada; 2 Department of Paediatrics and Adolescent Medicine, School of Clinical Medicine, Li Ka Shing Faculty of Medicine, The University of Hong Kong, Hong Kong SAR, China; 3 Pediatric Allergy & Asthma, Department of Pediatrics, University of Alberta, Edmonton, AB Canada; 4 Food, Nutrition, and Health, Faculty of Land and Food Systems, University of British Columbia, Vancouver, BC Canada; 5 Department of Pediatrics and Child Health, Section of Allergy and Immunology, University of Manitoba, Winnipeg, Canada

**Keywords:** Food ladders, Food allergy, Cow’s milk allergy, Egg allergy, Oral immunotherapy

## Abstract

**Background:**

Food ladders are tools designed to facilitate home-based dietary advancement in children with food allergies through stepwise exposures to increasingly allergenic forms of milk and egg. Several studies have now documented safety and efficacy of food ladders. In 2021, we published a Canadian adaptation of the previously existing milk and egg ladders originating in Europe using foods more readily available/consumed in Canada. Our study adds to the growing body of evidence supporting food ladder use and provides safety and effectiveness data for our Canadian adaptation of the milk and egg ladders.

**Methods:**

Surveys were distributed to families of children using the Canadian Milk Ladder and/or the Canadian Egg Ladder at baseline, with follow up surveys at 3 months, 6 months, and 12 months. Data were analyzed using REDCap and descriptive and inferential statistics are presented.

**Results:**

One hundred and nine participants were started on milk/egg ladders between September 2020 and June 2022. 53 participants responded to follow up surveys. Only 2 of 53 (3.8%) participants reported receiving epinephrine during the study. Severe grade 4 reactions (defined according to the modified World Allergy Organization grading system) were not reported by any participants. Minor cutaneous adverse reactions were common, with about 71% (n = 10/14) of respondents reporting cutaneous adverse reactions by 1 year of food ladder use. An increasing proportion of participants could tolerate most foods from steps 2–4 foods after 3, 6, and 12 months of the food ladder compared to baseline.

**Conclusion:**

The Canadian food ladders are safe tools for children with cow’s milk and/or egg allergies, and participants tolerated a larger range of foods with food ladder use compared to baseline.

**Supplementary Information:**

The online version contains supplementary material available at 10.1186/s13223-023-00847-7.

## Background

Cow’s milk and hen’s egg immunoglobulin E (IgE)-mediated food allergy (henceforth referred to as milk and egg allergy) are among the most prevalent allergies in childhood. The prevalence of these allergies in Canadian children is estimated at around 1.8 and 1.2 percent for milk and egg, respectively [1]. While the overall prognosis of both milk and egg allergy in children is generally favourable due to the relatively high likelihood of resolution, they can have a profound impact on quality of life and nutrition in young children, and avoidance of these ubiquitous foods can be difficult with accidental exposures being common [2–9]. Additionally, accurate laboratory predictors of resolution are lacking, and the diagnosis of resolution is dependent on oral food challenges which patients may have difficulty accessing in the real-world outside of controlled, research settings [10].

Food ladders are tools designed to guide a stepwise reintroduction of food allergens from extensively heated (i.e., baked) to less heated forms of protein. The advantages of using food ladders in the management of egg and milk allergic children is to facilitate home-based dietary expansion and encourage more rapid resolution of the food allergy. The first published food ladders originated in Europe and were originally intended for the management of non-IgE-mediated food allergy [11]. Several iterations of the milk and egg ladders have since been published for the management of IgE-mediated milk and egg allergies, including our Canadian Food Ladders in 2021, which adapted concepts from the European food ladders using foods readily available in the Canadian context (Supplementary materials 1 and 2) [12–17]. A handful of small studies have now been published to date on safety and efficacy of egg and milk ladders, utilizing European or Australian versions of the food ladders [13–15, 17–19]. This study sought to report the safety of the Canadian Food Ladders and to document dietary expansion through food ladder use, adding to the small but growing body of evidence supporting food ladders in the management of IgE-mediated milk and egg allergy.

## Methods

### Ethics approval

was granted through the UBC C&W Research Ethics Board. The previously used European milk and egg ladders were adapted by our team with foods more readily available/consumed in Canada [12]. Foods included and their order of appearance on the ladders were inspired by existing food ladders, and modifications were agreed upon by consensus opinion and best available evidence. Food ladders were then provided to Canadian allergists electronically and also distributed through the Canadian Society of Allergy and Clinical Immunology for more widespread participation. A participation link and QR code were embedded on the food ladders as well as on a coversheet, and versions of the ladders were provided in French and English. Participating allergists determined patient suitability to receive a food ladder for the advancement of diet as per typical individual practice. Included patients were under 18 years of age with allergist-diagnosed egg or milk allergy. Participation was voluntary and consent was obtained from participants (parents of children who were prescribed a food ladder), followed by a baseline survey establishing patient demographics such as age, atopic history, and foods tolerated prior to commencing ladder use (Supplementary materials 3). Follow up surveys were then distributed by email at 3, 6, and 12 months, assessing whether the patients were still using the Canadian Food Ladders, parental report of adverse reactions, symptoms associated with adverse reactions, and whether epinephrine was administered. Reactions were graded according to modified World Allergy Organization grading system [20]. A quality improvement approach was adopted, with regular monitoring of data for any concerning safety signals, and ability to improve our ladders in response to feedback. Data were analyzed using REDCap data software. Descriptive (e.g. frequencies) and inferential statistics were utilized.

## Results

One hundred and nine parents (109) of children using food ladders completed our baseline survey between September 2020 and June 2022. Fifty-three participants completed any follow up survey, with 44, 35, and 14 parents completing the 3-month, 6 month, and 12 months follow up surveys, respectively. The mean age of children using a food ladder was 3 years 2 months, ranging from 7 months of age to 15 years. Thirty children received the Canadian Milk Ladder, 63 received the Canadian Egg Ladder, and 16 received both ladders (Table 1). All patients reported confirmed sensitization to milk and/or egg with skin prick testing or positive allergen-specific IgE.

**Table 1:** Baseline patient characteristics

Baseline characteristic	Number of patients (frequency)
Mean age	3 years 2 months (38 months)
Median age	1 year 3 months (15 months)
Interquartile range	3 years 6 months (42 months)
Milk ladder	30/109 (27.5%)
Egg ladder	63/109 (57.8%)
Milk and egg ladder	16/109 (14.7%)
Underwent oral food challenge	
Milk	8/45 (17.8%)
Egg	15/78 (19.2%)
History of epinephrine use for milk and/or egg adverse reactions	
Yes	29/109 (26.6%)
No	79/109 (72.4%)
Unknown	1/109 (0.9%)
Comorbid food allergies	
Yes	51/109 (46.8%)
No	57/109 (52.3)
Unknown	1/109 (0.9%)
Peanut	42/109 (38.5%)
Tree nut	25/109 (22.9%)
Shellfish	6/109 (5.5%)
Sesame	5/109 (4.6%)
Fish	4/109 (3.7%)
Wheat	4 /109(3.7%)
Soy	2/109 (1.8%)
Other	14/109 (13.0%)
Asthma	
Yes	15/109 (13.8%)
No	66/109 (60.6%)
Unknown	28/109 (25.7%)
Allergic rhinoconjunctivitis	
Yes	22/109(20.2%)
No	59/109 (54.1%)
Unknown	28/109 (25.7%)
Eczema	
Yes	74/109 (67.9%)
No	7/109 (6.4%)
Unknown	28/109 (25.7%)
Eosinophilic esophagitis	
Yes	2/109 (1.8%)
No	79/109 (72.5%)
Unknown	28/109 (25.7%)

46.8% of participants reported other food allergies, with peanut allergy being the most common. Atopic comorbidities were common, with eczema reported in 67.9% of children, asthma in 13.8%, and allergic rhinoconjunctivitis in 20.2%.

**Fig. 1:**
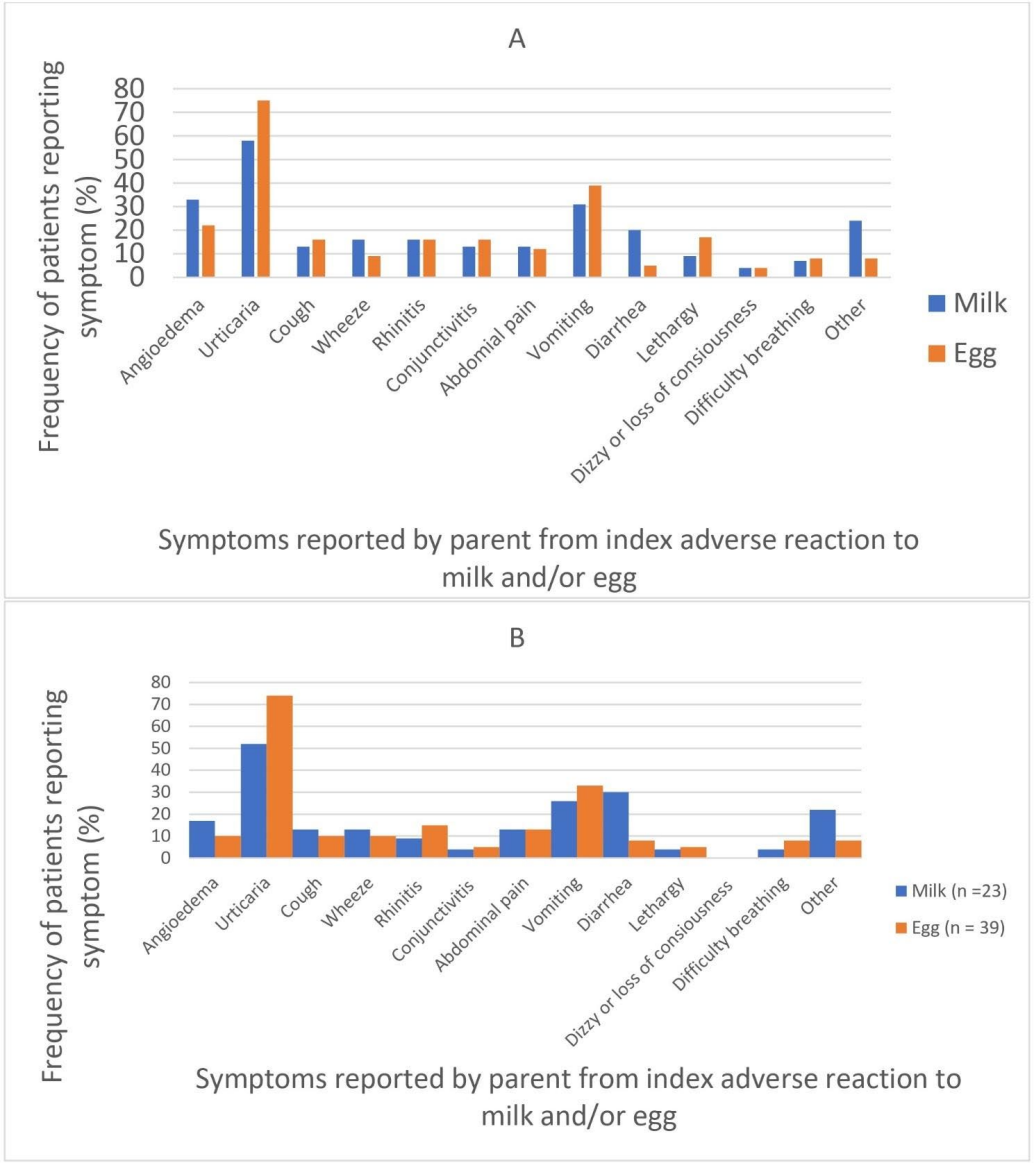
(A) Clinical symptoms reported by parent at index adverse reaction to milk and/or egg of entire baseline study population. (B) Clinical symptoms reported by parent at index adverse reacion to milk and/or egg for patients who completed follow up surveys

Symptoms reported at baseline with initial adverse reaction to milk and egg were similar, with the most common two symptoms reported being hives and angioedema (58% and 33% of patients reported hives and swelling, respectively, at initial reaction to milk, and 75% and 21% reported hives and swelling at initial reaction to egg) (Fig. 1A). 26.6% of all patients reported any history of epinephrine use due to adverse reactions to milk and/or egg. The population of participants who completed follow up surveys reported similar symptoms at initial adverse reaction to milk and/or egg compared to the entire study population that completed baseline surveys. 12/53 (22.6%) of participants who completed follow up surveys reported any history of epinephrine use due to adverse reactions to milk and/or egg.

**Fig. 2:**
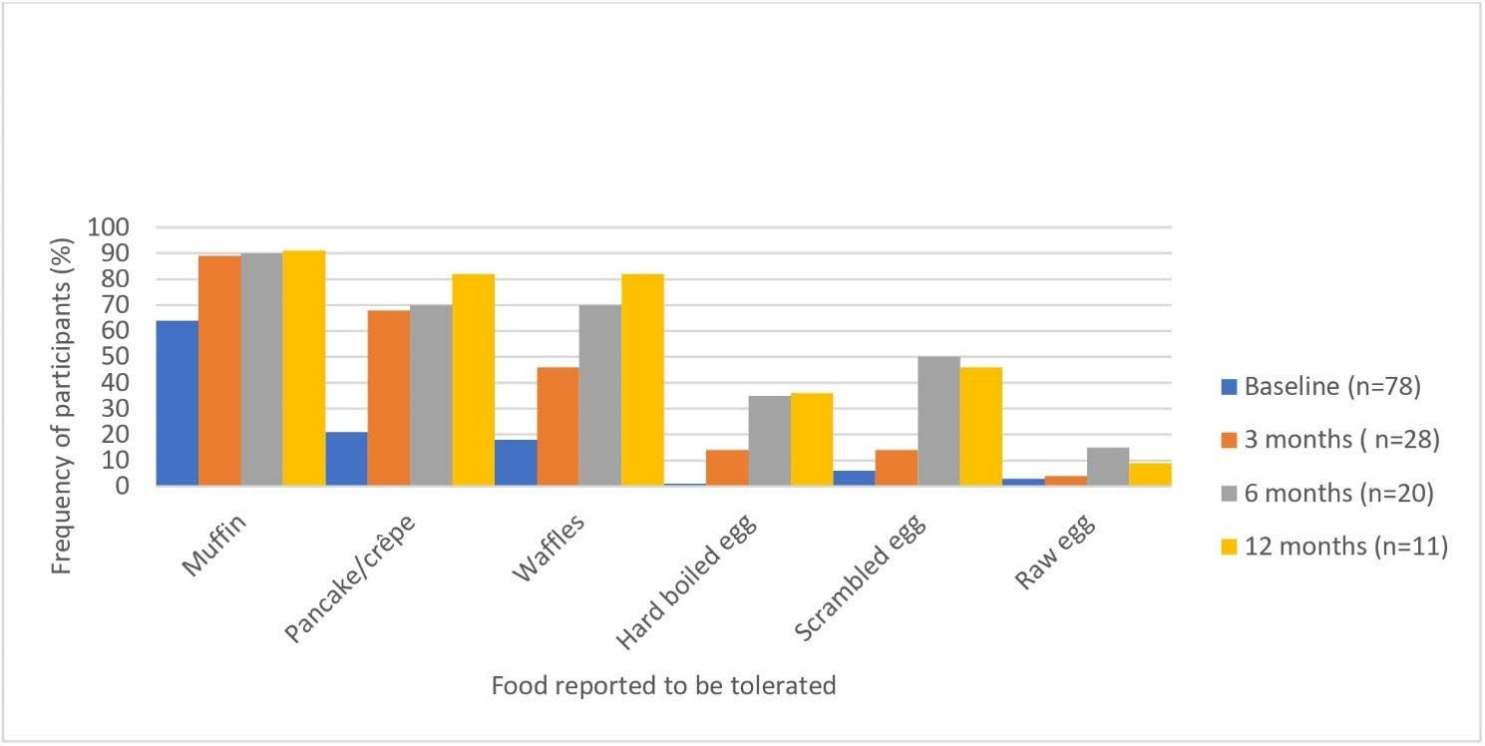
Proportion of patients reporting tolerance to foods from the egg ladder at baseline compared to at 3, 6, and 12 month follow up

**Fig. 3:**
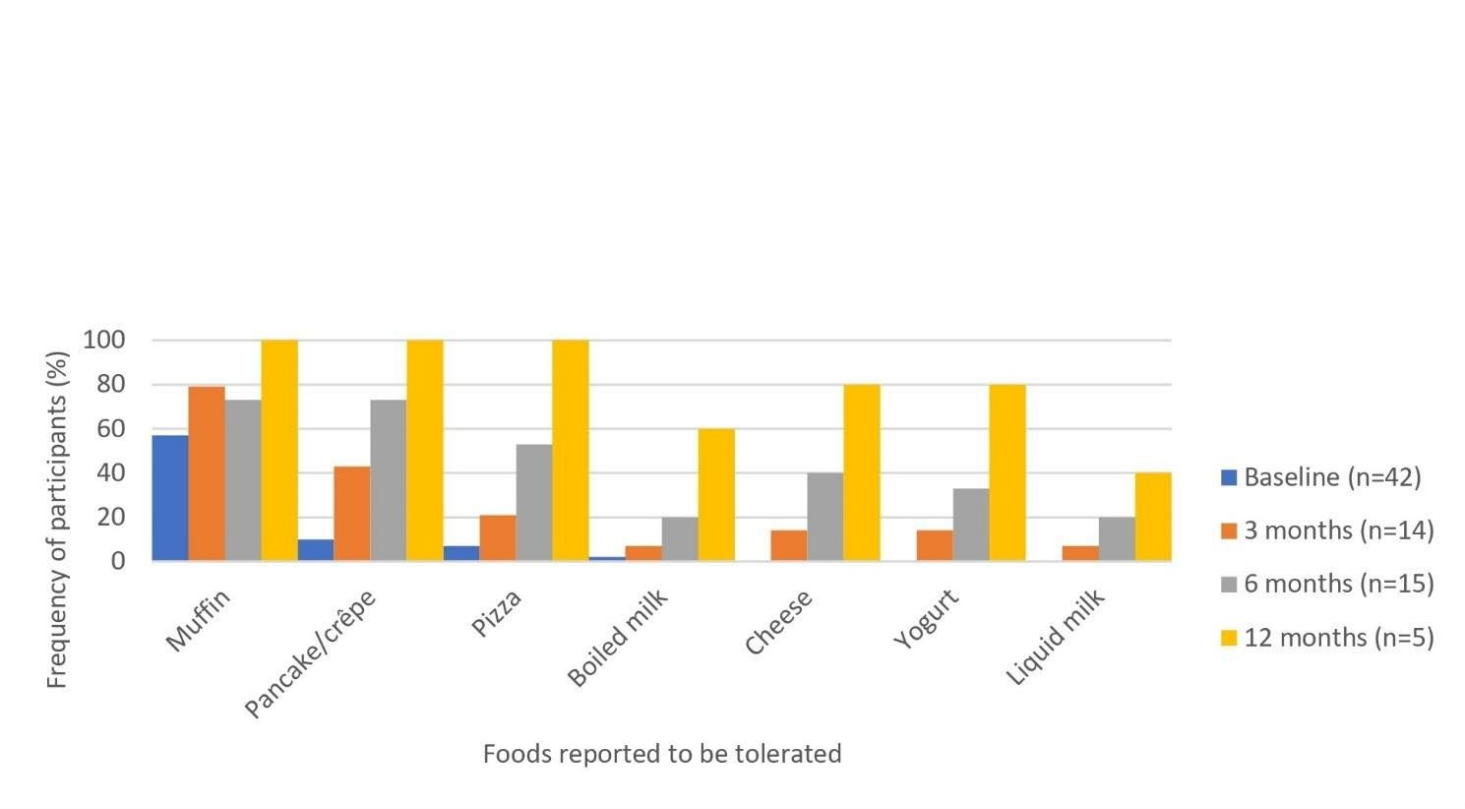
Proportion of patients reporting tolerance to foods from the milk ladder at baseline compared to at 3, 6, and 12 month follow up

At baseline, two-thirds (66.7%) of participants were tolerating at least one food from the milk ladder, and nearly three-quarters (73.3%) of participants were tolerating any food from the egg ladder (Fig. 2 and Fig. 3).

Increasing proportions of patients tolerated most step 2–4 foods at each follow up assessment. Isolated cutaneous adverse reactions were the most common adverse effect reported. About one fifth of patients reported any extracutaneous symptoms (Table 2). Severe Grade 4 reactions, defined by the modified WAO grading system, were not reported by any participants. Two of the 53 patients who completed follow up surveys (3.8%) indicated that a patient received epinephrine during the study. Both participants were egg allergic and experienced anaphylaxis while ingesting egg as per the egg ladder. Ages of the participants were 9 and 14 years old. One of these patients reported anaphylaxis with cough, rhinitis, and abdominal pain on the first day of ladder use to baked egg. The second patient reported anaphylaxis with cough, rhinitis, wheeze, conjunctivitis, hives, and angioedema to raw egg at 12 months follow up. Ten patients reported discontinuing using the food ladder (18.9%), 5 (9.4%) of which reported the reason for stopping was due to symptoms developing when eating foods from the ladder. Two patients did not specify a reason. One patient indicated that the ladder was too “difficult”- this is presumably related to adherence but was not specifically stated. Two patients were told by their physician to stop - this could be related to adherence or symptoms developing, but no specific reason given.

**Table 2:** Adverse events associated with food ladder use reported by parents at 3, 6, and 12 months follow up

Adverse event	Frequency reported at 3 months (N = 44)	Frequency reported at 6 months (N = 35)	Frequency reported at 12 months (N = 14)
Isolated cutaneous symptoms	7/44 (15.9%)	11/35 (31.4%)	10/14 (71.4%)
Any extracutaneous symptoms	10/44 (22.7%)	7/35 (20%)	3/14 (21.4%)
Cough	2/44 (4.5%)	0 (0%)	1/14 (7.1%)
Rhinitis	2/44 (4.5%)	0 (0%)	3/14 (21.4%)
Conjunctivitis	1/44 (2.2%)	1/35 (2.9%)	2/14 (14.3%)
Wheeze	0 (0%)	1/35 (2.9%)	1/14 (7.1%)
Vomiting	2/44 (4.5%)	4/35 (11.4%)	2/14 (14,3%)
Abdominal pain	6/44(13.6%)	3/35 (8.6%)	1/14 (7.1%)
Diarrhea	2/44 (4.5%)	2/35 (5.7%)	0 (0%)
Lethargy	0 (0%)	0 (0%)	0 (0%)
Dizziness/loss of consciousness	0 (0%)	0 (0%)	0 (0%)
Difficulty breathing	0 (0%)	0 (0%)	0 (0%)
Other symptoms	3/44 (6.8%)	3/35 (8.6%)	4/14 (28.5%)
Epinephrine administered	1/44 (2.3%)	0 (0%)	1/14 (7.1%)
Emergency room visit	1/44 (2.3%)	0 (0%)	1/14 (7.1%)

## Discussion

Our data support food ladder use for the management of IgE-mediated milk and egg allergy in preschoolers. An increasing proportion of respondents generally tolerated foods from step 2–4 in each follow up assessment.

Our effectiveness data is relatively similar to outcomes suggested by previous studies. D’Art et al. published a study reporting on efficacy of cow’s milk ladders, and reported 65% of children who used a milk ladder were tolerating cooked cheese (lasagna) at 6 months (vs 53% of our cohort tolerating cooked cheese on pizza by 6 months), and 82% tolerating lasagna at 12 months (vs 100% in our cohort) [18]. D’Art et al found 54% of children in their study tolerated pasteurized milk or powdered infant formula by 12 months of ladder use (vs 40% in our cohort) [18]. Cotter et al reported 41% of their cohort tolerated scrambled egg at 6 months (vs 50% in our cohort), and 69% (vs 46% in our cohort) at 12 months using the Irish Food Allergy Network (IFAN) egg ladder [15].

Similar to preschool oral immunotherapy, we found mild cutaneous symptoms occurred fairly frequently in our cohort [21]. Isolated cutaneous symptoms occurred in 31.4% of respondents at 6 months, and 71.4% of respondents by 1 year. Epinephrine was administered to two patients for symptoms of anaphylaxis during our study. The two patients who received epinephrine were 9 and 14 years of age. One of the two patients who received epinephrine had a history of prior anaphylaxis to egg. Neither patient reported symptoms suggestive of severe grade 4 anaphylaxis as per the modified WAO grading system. Our initial Canadian food ladder publication already had stated that the ladders were intended for use in preschoolers with a history of mild reactions, but since a small subset of the surveys showed that patients with a history of anaphylaxis and/or those of school age were started on ladders, we decided to publish our proposed Food Ladder Safety Checklist 4 A’s (age, active or poorly controlled asthma, history of anaphylaxis, and adherence) to reinforce this further [12, 22]. The older age of the two patients who experienced anaphylaxis supports our recommended criteria for patient selection. No patients who met all 4 A’s were treated for anaphylaxis [22].

Compared to other existing ladders, the Canadian Food Ladders are relatively similar in terms of types of foods included and order of appearance. A decision was made to remove butter from the Canadian Milk Ladder due to variable and typically very low cow’s milk protein content across available butter products. Recently, De Vlieger et al suggested that boiled egg may be introduced earlier on the egg ladder, prior to pancakes and waffles based on lower ovalbumin content of hard-boiled egg [17]. In their cohort of 78 children, reported rates of adverse reactions were relatively low, however the largest proportion of adverse events occurred with hard-boiled egg ingestion in their study. In contrast, the foods most commonly reported to be associated with adverse reactions in our study was baked milk and egg, potentially attributable to the fact that the largest number of respondents consumed these foods during our study.

Our study had several limitations. Attrition was high by 12 months, making response bias a concern. However, the response rate at 3 and 6 months was satisfactory for a real-world setting outside of a controlled research environment. Similar studies suggest that many children may outgrow milk/egg allergies after longer than 12 months of ladder use, and therefore we were unable to capture complete course of treatment for our cohort. Additionally, epinephrine administration was used as an indicator of anaphylaxis, and anaphylaxis that was not appropriately treated with epinephrine would not be accounted for in our data. Finally, oral food challenges were not conducted at follow-up, and therefore effectiveness data is restricted to parental reports of tolerance of specific foods which may result in reporting bias due to variation in symptom perception. Strengths of our study included our real-world approach. Ladders were prescribed and managed by a wide range of allergists from across Canada, as per the prescribing allergists’ typical practice. Ongoing monitoring allowed us to make adjustments for safety and improve content, including the addition of the safety checklist and translation of the ladders from English to French.

Further research continues to be necessary for patients with IgE-mediated milk and/or egg allergies managed using food ladders. A larger sample size with control group or randomized controlled trial would be valuable to add strength to the results of this study. Kim et al. examined induction of sustained unresponsiveness in young children (mean age 7.3 years) who were tolerant to baked egg but not unbaked egg following 2 years of treatment with either egg OIT or with baked egg consumption. They found a clear benefit of OIT over baked egg consumption only with only 11.1% of baked egg tolerant children achieving sustained unresponsiveness compared to 43.5% of baked egg reactive participants who received OIT [23]. It is unclear at this point how food ladders might compare to OIT as no randomized controlled trials exist comparing oral immunotherapy (OIT) to food ladders for safety, efficacy, or quality of life measures. Whether food ladders lead to a permanent state of tolerance has also yet to be established and long term follow up data is required.

Food ladders as management tools for IgE-mediated milk and egg allergies in young children have the potential to ease health care resource utilization through reducing the need for oral food challenges and potentially offering a home-based alternative for oral immunotherapy for some children. In addition, food ladders can offer flexibility and allows for more natural consumption of milk and/or egg compared to oral immunotherapy and may provide a lower cost alternative to oral immunotherapy in some circumstances. Our study reinforces the importance of appropriate patient selection for food ladder use, particularly with older age being a potential risk factor for adverse reactions.

## Conclusions

Our study adds to a growing body of evidence supporting milk and egg ladder use. Our safety outcomes were similar to data published on previously existing European milk and egg ladders. Isolated cutaneous symptoms associated with food ladder use are relatively common, with severe symptoms or epinephrine administration being uncommon. In our cohort, both patients who received epinephrine were over the age of 6 years old, which supports our previously proposed criteria for patient selection for food ladder use. Therefore, our study supports the safety and appropriateness of milk and egg ladder use in those patients who meet the stringent criteria outlined in the 4As. Until further study can be completed revealing safety in other groups, including older patients, we caution against using these ladders in any patient who does not meet those stringent criteria

### Electronic Supplementary Material

Below is the link to the electronic supplementary material

Supplementary Material 1: Canadian Milk Ladder

Supplementary Material 2: Canadian Egg Ladder

Supplementary Material 3: Baseline survey

Supplementary Material 4: Follow up survey

## Data Availability

The datasets used and/or analysed during the current study are available from the corresponding author on reasonable request.

## Abbreviations

IgE: Immunoglobulin E
OIT: Oral immunotherapy
