# Supplementary figures and images for: Safety and effectiveness of the Canadian food ladders for children with IgE-mediated food allergies to cow’s milk and/or egg
Source: Allergy Asthma Clin Immunol. 2023 Nov 6;19:94. doi: 10.1186/s13223-023-00847-7 (PMC10629013; doi:10.1186/s13223-023-00847-7)

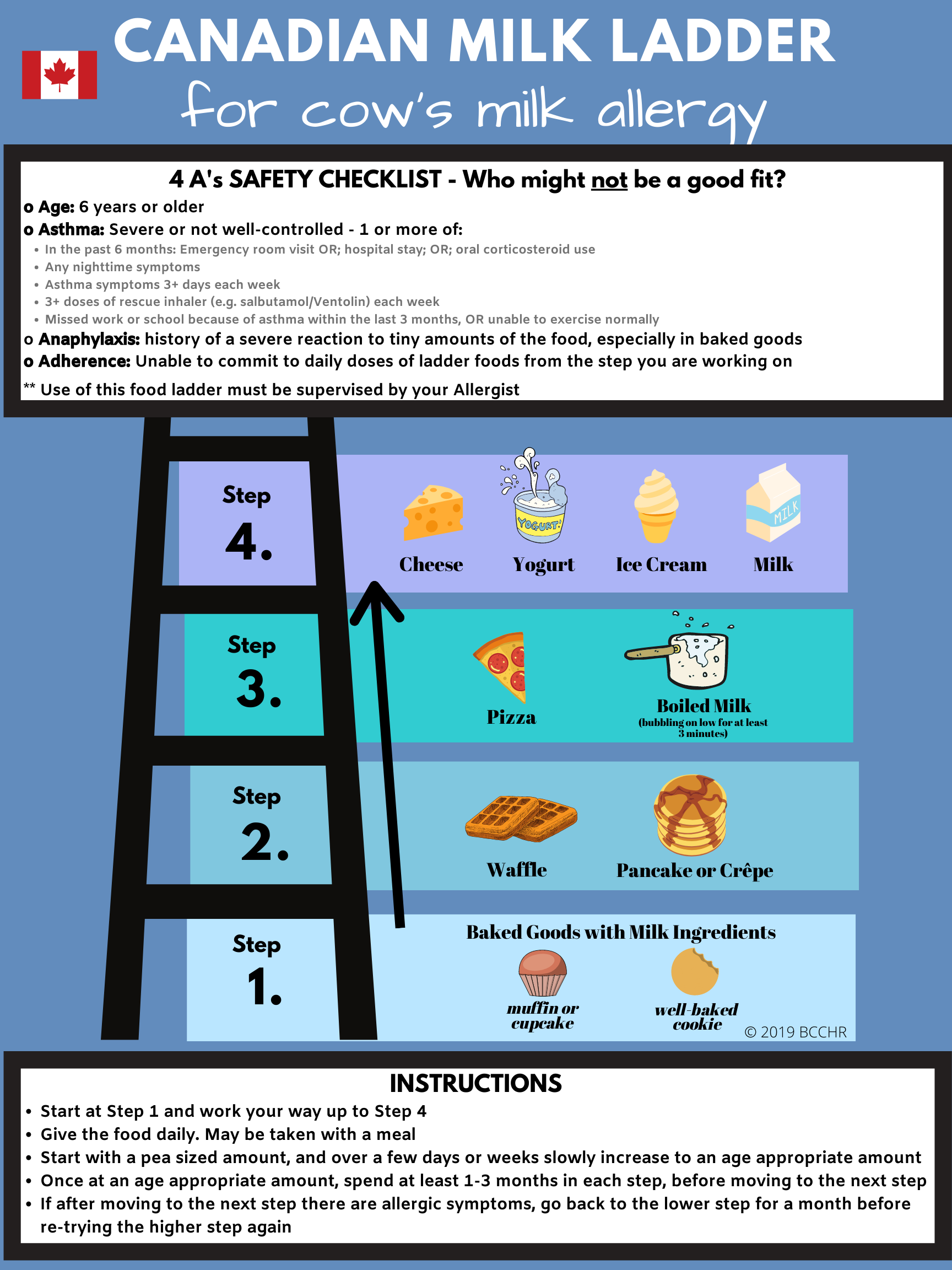

Supplement: Supplementary file 1 — Supplementary Material 1: Canadian Milk Ladder [file 13223_2023_847_MOESM1_ESM.png]

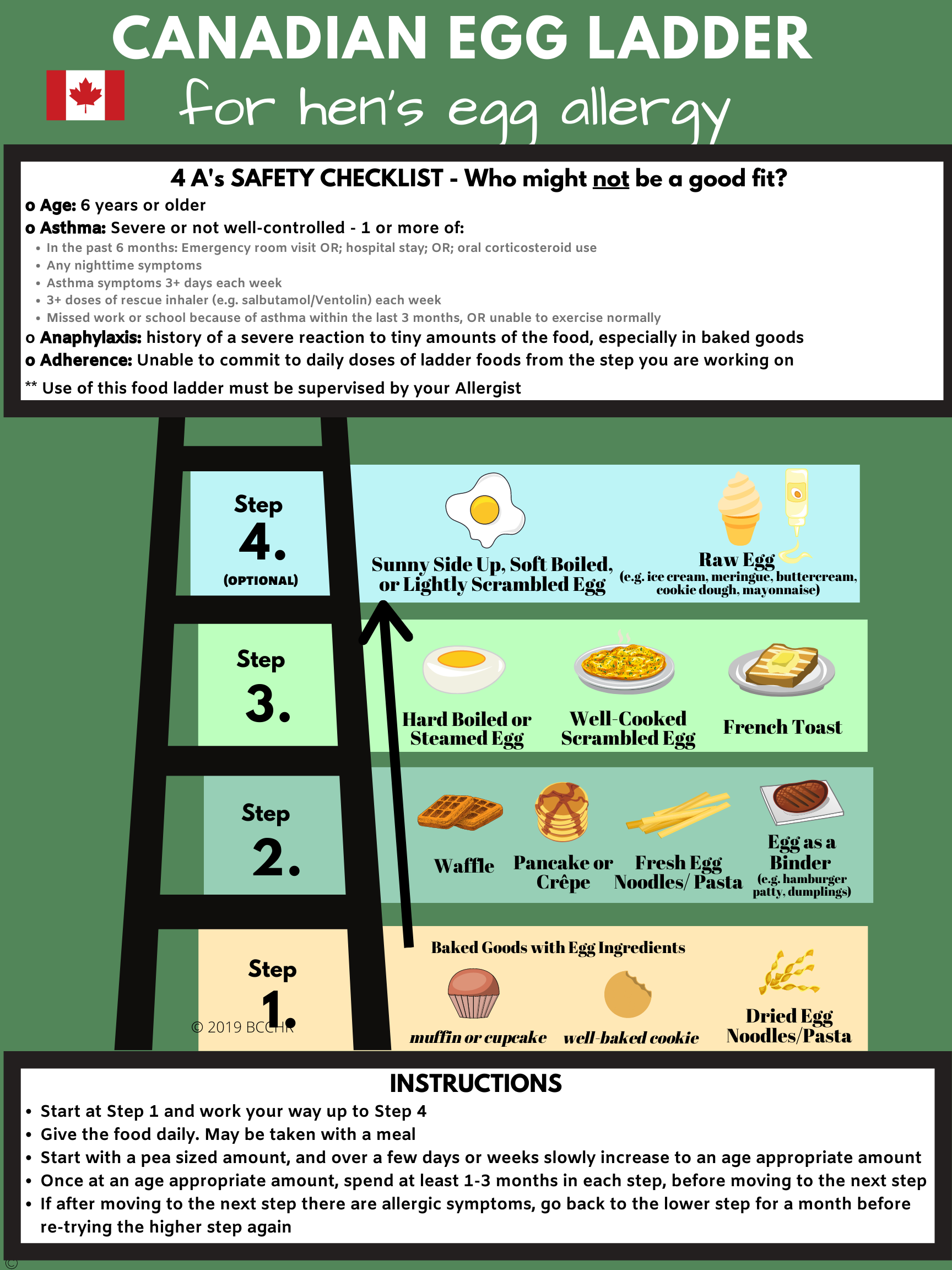

Supplement: Supplementary file 2 — Supplementary Material 2: Canadian Egg Ladder [file 13223_2023_847_MOESM2_ESM.png]
