# Supplementary material for: Safety and effectiveness of the Canadian food ladders for children with IgE-mediated food allergies to cow’s milk and/or egg
Source: Allergy Asthma Clin Immunol. 2023 Nov 6;19:94. doi: 10.1186/s13223-023-00847-7 (PMC10629013; doi:10.1186/s13223-023-00847-7)
Supplement: Supplementary file 3 — Supplementary Material 3: Baseline survey [file 13223_2023_847_MOESM3_ESM.pdf]

**Milk/Egg Ladder Survey for Parents- Baseline survey**

1. How old is your child?
   ____ years ___months

2. Which food ladder(s) has been provided for your child?
   a. Milk
   b. Egg
   c. Both

*Branching logic from Q2 onwards

3. When your child had his/her first reaction to milk/egg, which of the following occurred (choose all that apply):
   a. Swelling of the lips/eyes/tongue/face
   b. Hives
   c. Cough
   d. Wheeze
   e. Runny, itchy, congested nose, and/or sneezing
   f. Red, watery, and/or itchy eyes
   g. Abdominal pain
   h. Vomiting
   i. Diarrhea
   j. Very sleepy or quiet
   k. Dizzy or passing out
   l. Difficulty breathing
   m. Other (Free text)

4. How old was your child at the time of his/her first reaction to milk/egg?
   ___ years ____ months

5. Which of the following did your child have for milk/egg prior to starting on the food ladder? (Choose all that apply)
   a. Positive skin prick test
   b. Positive blood test
   c. Positive food challenge
   d. None

6. Has your child ever been given epinephrine (EpiPen) for an allergic reaction to milk/egg?
   a. Yes
      7b. Does your child have an epinephrine autoinjector with them at all times?
         i. Yes
         ii. No

b. No

7. At which category of foods on the milk/egg ladder did your allergist recommend starting your child?
    a. Step 1 foods (Baked goods with milk ingredients/ baked goods with egg ingredients, dried egg noodles,)
    b. Step 2 foods (Pancakes, crêpes, waffles/pancakes, crêpes, egg as a binder in hamburger, dumplings etc., waffles, fresh egg noodles)
    c. Step 3 foods (Pizza, boiled milk/Hard boiled or steamed egg, well-cooked scrambled egg, French toast)
    d. Step 4 foods (Milk, yogurt, cheese, ice cream/ lightly scrambled egg, soft boiled egg, sunny side up egg, raw egg)

8. Which foods was your child already tolerating when they started using the milk/egg ladder?
    a. Yes (Choose all that apply)
        i. Muffin or cupcake
        ii. Well-baked cookie
        iii. Pancakes/crêpes
        iv. Waffles
        v. Pizza
        vi. Boiled milk
        vii. Cheese
        viii. Yogurt
        ix. Ice cream
        x. Milk

        xi. Baked goods with egg ingredients
        xii. Dried egg noodles
        xiii. Pancakes/ crêpes
        xiv. Waffles
        xv. Fresh egg noodles/pasta
        xvi. Egg as a binder in hamburger patty, dumplings etc.
        xvii. Hard-boiled or steamed egg
        xviii. Well-cooked scrambled egg
        xix. French toast
        xx. Lightly scrambled egg/soft boiled egg/sunny side up egg
        xxi. Raw egg (ice cream, meringue, mayonnaise, buttercream, cookie dough etc.)
        xxii. None

    b. No

9. Has your child been diagnosed with food allergies other than to milk/egg?
    a. Yes
        i. Which other foods are your child allergic to?
            a) Peanut
            b) Almond
            c) Hazelnut
            d) Cashew
            e) Pistachio
            f) Walnut
            g) Pecan
            h) Sesame
            i) Shellfish
            j) Fish
            k) Wheat
            l) Soy
            m) Other (Free text)
    b. No

10. Does your child have any of the following conditions? (Choose all that apply)
    a. Asthma
       10b) Has your child been to the emergency room in the last 1 year for asthma symptoms?
            a) Yes
            b) No
    b. Environmental allergies
    c. Eosinophilic esophagitis
    d. Eczema

11. Does your child have any first-degree relatives (biological parents/siblings) with food allergies, environmental allergies, eczema or asthma?
    a. Yes
    b. No

Thank you for participating in our study.
