# Supplementary material for: Safety and effectiveness of the Canadian food ladders for children with IgE-mediated food allergies to cow’s milk and/or egg
Source: Allergy Asthma Clin Immunol. 2023 Nov 6;19:94. doi: 10.1186/s13223-023-00847-7 (PMC10629013; doi:10.1186/s13223-023-00847-7)
Supplement: Supplementary file 4 — Supplementary Material 4: Follow up survey [file 13223_2023_847_MOESM4_ESM.pdf]

Milk/Egg Ladder Survey for Parents- 3, 6, 12-month survey

1. Is your child still eating foods from the egg/milk ladder?
    a. Yes
    b. No
    1b. If not, why was the ladder stopped?
        1. Allergy symptoms developed when eating foods from the ladder
        2. Eating food from the ladder was causing worry
        3. I was told by a doctor to stop using the food ladder
        4. My child did not want to continue
        5. It was too difficult to continue with the food ladder
        6. The food ladder was not applicable to my child
        7. My child has outgrown his/her allergy
        8. Other

    1c. If your family has stopped using the food ladder for your child, how long did you use the ladder for prior to stopping?
        1. We decided not to start
        2. 1 day
        3. Less than 7 days
        4. 7 days- less than 30 days
        5. 1 months – less than 3 months
        6. 3 months - less than 6 months
        7. More than 6 months

2. Which foods on the food ladder is your child currently tolerating (choose all that apply)?
    i. Muffin or cupcake
    ii. Well-baked cookie
    iii. Pancakes/crêpes
    iv. Waffles
    v. Pizza
    vi. Boiled milk
    vii. Cheese
    viii. Yogurt
    ix. Ice cream
    x. Milk

    xi. Baked goods with egg ingredients
    xii. Dried egg noodles
    xiii. Pancakes/ crêpes
    xiv. Waffles
    xv. Fresh egg noodles/pasta
    xvi. Egg as a binder in hamburger patty, dumplings etc.
    xvii. Hard-boiled or steamed egg

xviii. Well-cooked scrambled egg
xix. French toast
xx. Lightly scrambled egg/soft boiled egg/sunny side up egg
xxi. Raw egg (ice cream, meringue, mayonnaise, buttercream, cookie dough etc.)
xxii. None

3. For how long (on average) have you been feeding each category of food on the milk/egg ladder before moving to the next category?
   a. Less than 1 week
   b. 1 week - 2 weeks
   c. 2 weeks – 4 weeks
   d. 1 month – 2 months
   e. 2 months – 4 months
   f. Longer than 4 months
   g. Other (free text)

4. How long do you feed a new food on the milk/egg ladder before introducing another food from the same category?
   a. Less than 1 week
   b. 1 week - 2 weeks
   c. 2 weeks – 4 weeks
   d. 1 month – 2 months
   e. 2 months – 4 months
   f. Longer than 4 months
   g. Other (free text)

5. Has your child experienced any skin reactions (for example: hives or rash) as a result of eating foods on the food ladder?
   a. Yes
   b. No

6. Has your child experienced any allergic reactions with symptoms other than on the skin (for example: vomiting, abdominal pain, swelling of the lips/eyes/tongue/face, cough, difficulty breathing) as a result using the food ladder?
   a. Yes
      6a. Which foods(s) were related to your child's allergic reaction when using the food ladder (free text)
      6b. Which symptoms has your child experienced as a result of using the food ladder? (Choose all that apply)
         a. Swelling of the lips/eyes/tongue/face
         b. Hives
         c. Cough
         d. Wheeze

e. Runny, itchy, congested nose, and/or sneezing
f. Red, watery, and/or itchy eyes
g. Abdominal pain
h. Vomiting
i. Diarrhea
j. Very sleepy or quiet
k. Dizzy or passing out
l. Difficulty breathing
m. Other

b. No

7. Since starting the food ladder has your child developed vomiting, difficulty swallowing foods, or feeling like food is getting stuck?
    a. Yes
    b. No

8. Have you given epinephrine (EpiPen) to your child because of any allergic reactions resulting from using the food ladder?
    a. Yes
    b. No

9. Have you brought your child to the emergency room because of any allergic reactions resulting from using the food ladder?
    a. Yes
    b. No

10. Has your child seen his/her allergist because of questions or concerns related to the food ladder?
    a. Yes
    b. No

11. Has your child seen a doctor other than his/her allergist for questions or concerns related the food ladder?
    a. Yes
    b. No

12. Do you feel safe progressing to a new category of foods on the ladder at home?
    a. Yes
    b. No
    c. Unsure

13. How easy to use is the food ladder?

a. Very difficult
b. Somewhat difficult
c. Neither difficult nor easy
d. Somewhat easy
e. Very easy

14. What was the most difficult part about using the food ladder in your everyday life?
    Free text

15. How many days per week is your child eating foods from the food ladder?
    a. 1- 2
    b. 3- 4
    c. 5- 6
    d. Everyday

16. How many hours per week do you spend preparing food for the food ladder?
    a. 1- 2
    b. 3- 4
    c. 4-5
    d. 5-6
    e. 7 hours or more

17. Would you prefer more examples of foods from each category on the ladder, or do you prefer the current level of guidance?

    a. Yes, more examples would be helpful
    b. No, I am happy with the current amount of guidance

18. Has your child seen his/her allergist since starting the food ladder?
    a. Yes
       18b. Did your child have a repeat skin prick test or blood test to the food on the food ladder?
       i. Yes
          18c. Did the skin prick test or blood test show that your child is still allergic?
          a. Yes, the test was positive
          b. No, the test was negative
          c. I don't know
       ii. No
       iii. I don't know
    b. No
